# Immunological characterisation of an acute murine model of lung inflammation and airway hyperreactivity

**DOI:** 10.1186/1476-9255-10-S1-P7

**Published:** 2013-08-14

**Authors:** Kate L Dixon, Yee-Man Ching, Julie Coote, Neil Gozzard

**Affiliations:** 1 UCB Pharma, Slough, Berkshire, SL1 3WE, UK

## 

Asthma is associated with chronic airway inflammation and airway hyperreactivity (AHR). Approximately 5-10% of the asthmatic patient population suffers from severe disease which remains uncontrolled by current therapies. An acute murine model that replicates aspects of severe asthma was established. Airway inflammation and AHR were elicited by s.c. sensitisation to house dust mite extract (HDM) in complete Freund’s adjuvant (CFA) and a subsequent HDM challenge into the airways (i.n.) 14 days later. The ensuing inflammation comprised of eosinophilia, a marked neutrophilia and a mixed T cell response with significant infiltration of Th1, Th2 and Th17 cells into airways. To investigate the role of these T-helper subsets in this model, the effects of neutralising the key T-helper cell cytokines IFNγ (Th1), IL-4 (Th2) and IL-17 (Th17) were evaluated.

Neutralising antibodies to IFNγ, IL-4 and IL-17 were administered prophylactically (s.c), from one day prior to HDM immunisation. AHR to methacholine was assessed by Penh 48 hours post HDM challenge followed by collection of broncho-alveolar lavage (BAL) and serum for analysis of cellular inflammation, cytokines and HDM specific antibodies.

IFNγ neutralisation profoundly inhibited AHR yet enhanced BAL eosinophilia, neutrophilia, IL-17 levels and HDM specific serum IgE. Conversely, anti-IL-4 treatment inhibited eosinophil accumulation, IL-13 production and HDM specific serum IgE and IgG1, without affecting AHR. Anti-IL-17 treatment reduced BAL neutrophilia and KC levels, however no significant modulation of AHR, eosinophilia or HDM specific serum IgE and IgG1 was observed.

In summary, the T cell cytokines IFNγ, IL-4, and IL-17 each play distinct roles in the development of airway inflammation and AHR in this acute murine model. Furthermore, these data indicate there is dissociation between cellular inflammation and hyperreactivity of the airways in this model. In conclusion, this acute HDM driven model may be representative of the mixed inflammatory response (Th1, Th2 & Th17) reported to be present in the airways of severe asthmatics.

